# Individual experience influences reconstruction of division of labour under colony disturbance in a queenless ant species

**DOI:** 10.1186/s12983-022-00466-9

**Published:** 2022-06-15

**Authors:** Yasunari Tanaka, Masaru K. Hojo, Hiroyuki Shimoji

**Affiliations:** School of Biological and Environmental Sciences, Kwansei Gakuin University, Sanda, Hyogo 669-1330 Japan

**Keywords:** Behavioural flexibility, Response threshold, Age, Self-organization, Hymenoptera

## Abstract

**Background:**

Division of labour (DOL) is ubiquitous across biological hierarchies. In eusocial insects, DOL is often characterized by age-related task allocation, but workers can flexibly change their tasks, allowing for DOL reconstruction in fluctuating environments. Behavioural change driven by individual experience is regarded as a key to understanding this task flexibility. However, experimental evidence for the influence of individual experience is remains sparse. Here we tested the effect of individual experience on task choice in the queenless ponerine ant, *Diacamma* cf. *indicum* from Japan.

**Results:**

We confirmed that both nurses and foragers shifted to vacant tasks when the colony composition was biased to one or the other. We also found that nurses which are induced to forage readily revert to nursing when reintroduced into balanced colonies. In contrast, foragers which are induced to revert to nursing very rarely return to a foraging role, even 19 days post reintroduction to their original colony.

**Conclusions:**

Taken together, our results suggest that individual experience decreases the response threshold of original foragers, as they continue to be specialist nurses in a disturbed colony. However, original nurses do not appear strongly affected by having forager experience and revert to being nurses. Therefore, while individual experience does have an effect, other factors, such as reproductive ability, are clearly required to understand DOL maintenance in fluctuating environments.

**Supplementary Information:**

The online version contains supplementary material available at 10.1186/s12983-022-00466-9.

## Background

Division of labour (DOL), or functional specialization among subpopulations, has evolved across all levels of biological organization [1–3]. A typical example of DOL is in the multicellular green alga *Volvox*, with one type of cell assigned to reproduction and different type to other tasks [4]. Group-living organisms across a wide range of taxa also exhibit DOL on the individual level. In particular, eusocial insects, such as honeybees, ants, and termites, have highly sophisticated social systems based on reproductive specialization of a queen and a non-reproductive worker caste [5]. These workers are further specialized to different tasks, and this DOL enhances colony productivity [6].

Individual and colony-level factors such as age, genotype, and social demands are involved in DOL formation among workers in social insects [7]. Generally, age is the primary factor determining worker behavioural caste [8]. Workers initially take care of the brood as nurses inside a nest, and switch from nursing to foraging outside the nest. This temporal polyethism is an adaptive strategy enhancing colony-level efficiency [9, 10]. However, the age structure of a colony also changes temporally, such as with the death of workers. Studies across several taxa have reported that workers shift flexibly from their current tasks to the other tasks depending on social demands; for example, foragers can shift back to the nurse role when the number of nurses is insufficient [8, 11–17]. Physiological factors necessary to the performance of the tasks also alter depending on that task [18–23]. Thus, worker behavioural flexibility maintains robustness of DOL in insect societies.

Individual experience, such as predator encounters and physical competitions, influences behavioural variation [24–29]. Fluctuating environmental conditions favour experience-based behavioural change [30]. In social insects, individual experience amplifies behavioural variation among workers, leading to group-level pattern formation, with subpopulations splitting tasks such as foraging, defence, and communication [31]. Additionally, a worker’s recent task experience is an important factor in whether that individual will perform a given task [7]. For example, Ravary et al. [32] reported that in the clonal raider ant (*Ooceraea biroi*), workers differentiated to foragers or nurses depending on previous task experience.

Such experience-based task specialization has been explained using a response threshold reinforcement model [33]. This model is based on the concept of a fixed response threshold [8, 34, 35], which suggests that each worker has internal thresholds to a cue associated with a task. Workers will perform the task when that cue exceeds the threshold, and once the task is complete, the stimulus decreases. Consequently, variation in this threshold causes differential behavioural responses of workers, leading to DOL. Through incorporating individual experience into this self-organizing process, the response threshold reinforcement model explains flexible behavioural change of workers in a disturbed colony [33]. The reinforcement model assumes that the response threshold itself changes depending on experience with a task. In a disturbed colony, the model predicts that the thresholds of task-shifted workers are reduced, leading to reorganization of DOL [33]. However, other factors besides response threshold could explain worker task shift [7, 14, 35–37]. For example, shifting from foragers to nurses in a colony with the nurses removed would not necessarily require a threshold change. To address the difficulty of empirically testing for threshold changes, Theraulaz et al. [33] proposed a behavioural experiment that directly compares the threshold. While several experimental studies demonstrated that workers in many species flexibly task-shift, few have tested the effect of task experience on this change (but see [11]).

In this study, we examined the relationship between individual experience and task choice in the queenless ponerine ant, *Diacamma* cf. *indicum* from Japan. The only *Diacamma* species endemic to Japan, *D.* cf. *indicum* is ideal for such an experiment. A colony comprises up to 300 workers and the gamergate [38], a worker that acts as a functional queen, mated with one male [39, 40]. Age generally determines DOL [41, 42], but when nurses are experimentally removed, foragers can revert to nurses within 1 day [15, 19]. These biological features allow us to test how individual experience at different tasks subsequently affects the response threshold of workers. Our results should improve current understanding of the mechanisms maintaining DOL under fluctuating environmental conditions.

## Results

### Effect of behavioural propensity on task choice

**Fig. 1:**
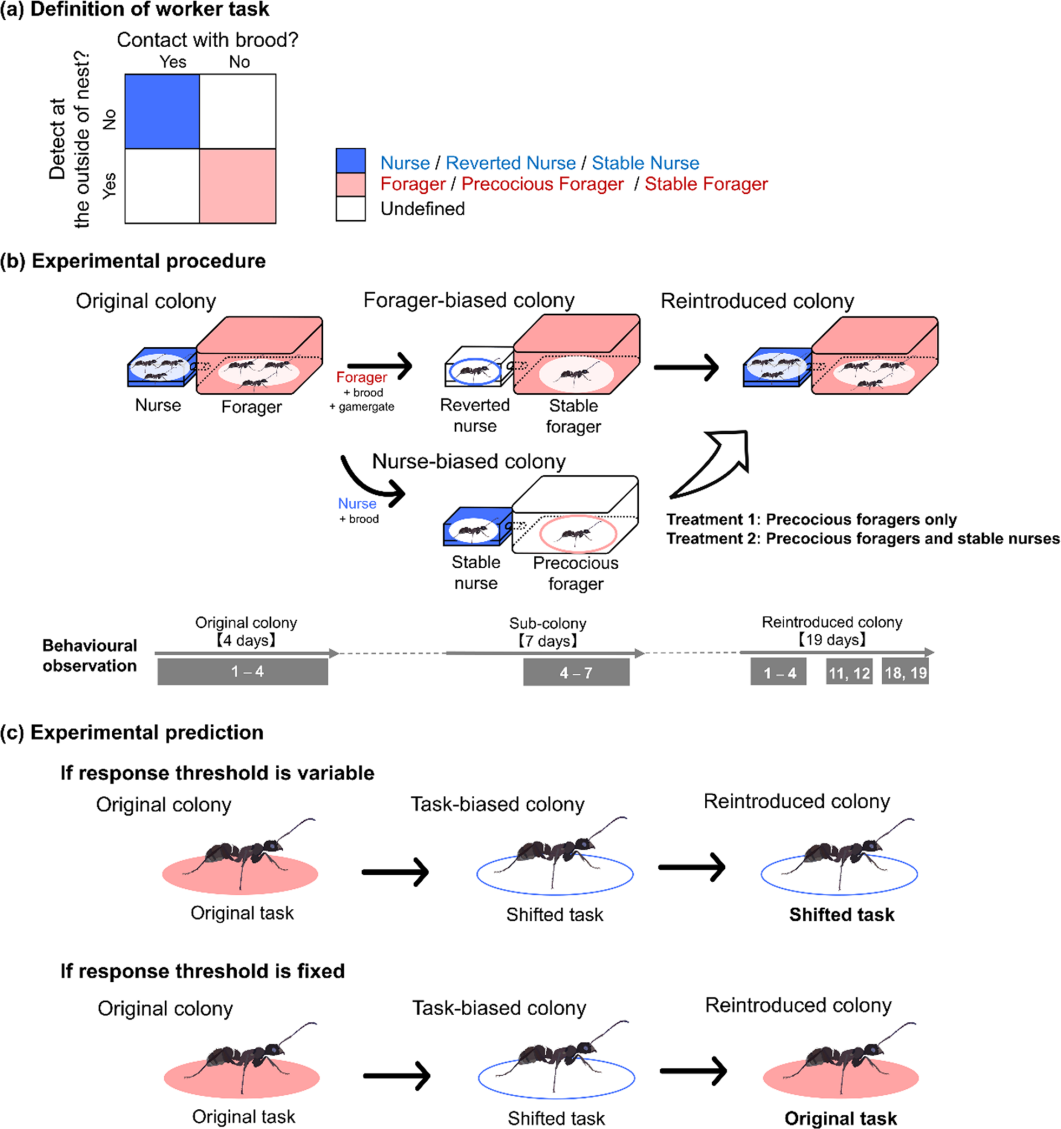
Task definition and experimental design. **a** Worker tasks were divided into three categories. **b** Foragers and nurses of a single colony were separated into two sub-colonies: the forager-biased colony and nurse-biased colony. After 7 days, workers from the nurse-biased colony were reintroduced into the forager-biased colony to form the reintroduced colony (treatment 1: precocious foragers were introduced; treatment 2: stable nurses and precocious foragers were introduced). **c** Interpretation of worker task choice in the reintroduced colony based on threshold models

**Fig. 2:**
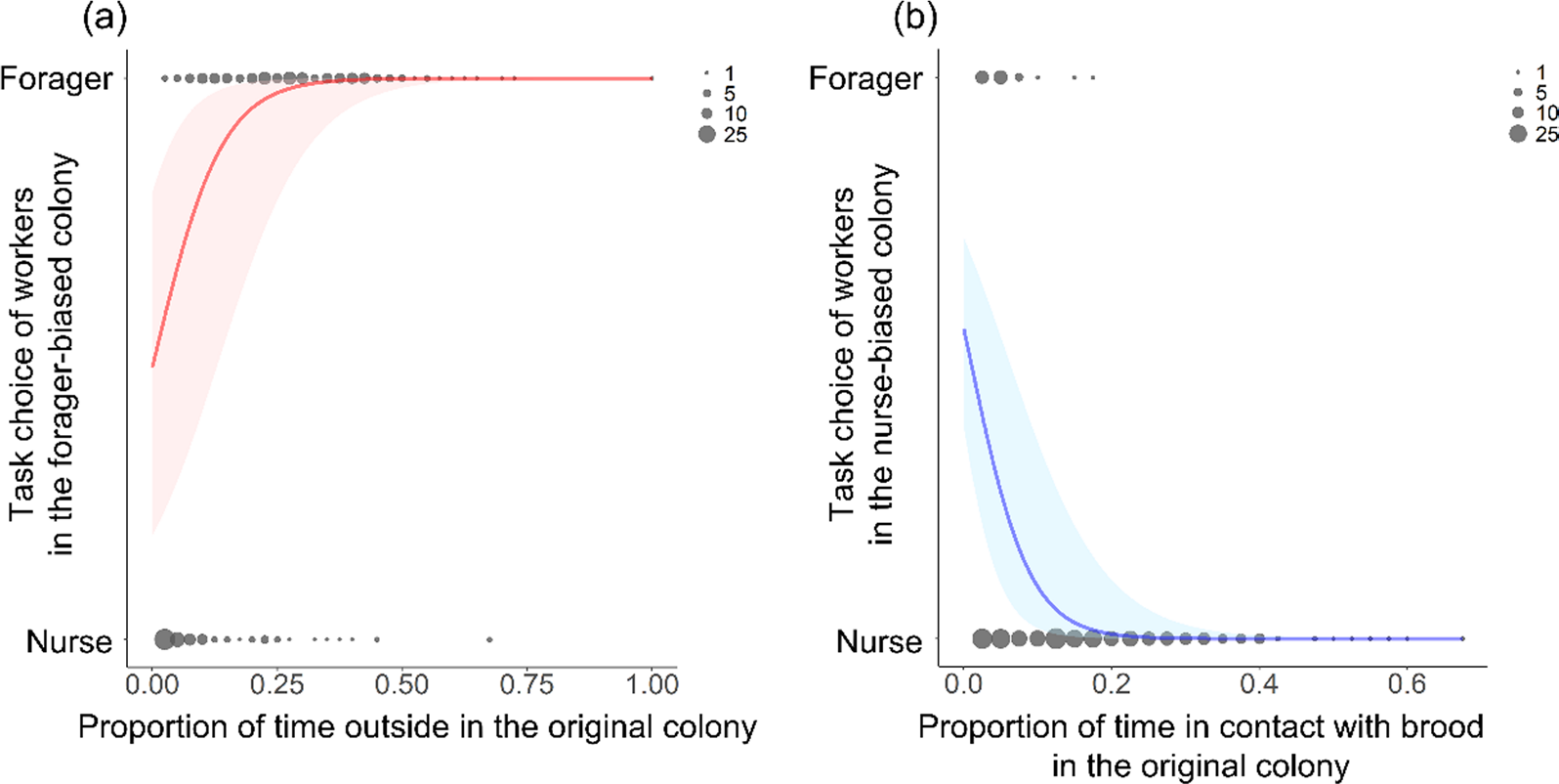
Relationship between task choice and behavioural propensity of workers. **a** Task choice of foragers in forager-biased colonies and **b** task choice of nurses in nurse-biased colonies. Circle sizes represent the number of overlapping data points. Lines and shades indicate estimated prediction and 95% confidence intervals, respectively

In a previous study, the foraging activity of foragers was found to be negatively correlated with the probability of becoming reverted nurses [15]. First, to validate previous results using the current observation method, we examined the effect of task frequency in the original colony on the task-choice of foragers in the forager-biased colonies (Fig. 1b). For this analysis, we used the ratio of foraging activity to the 40 observation times in the original colonies and worker tasks (the nurse or the forager) categorized based on 40 observation times in the forager-biased colonies (see “Method” section). In forager-biased colonies, foragers with little foraging activity in their original colony shifted to nurses as reverted nurses (GLMM: *χ*^2^ = 62.121, *P* < 0.001, *R*^2^ = 0.886; Fig. 2a, Additional file 1: Table S1). This result strongly supports the conclusions of our previous study [15] that recent activity as foragers influences subsequent task choice of forager in disturbed conditions. Second, using the ratio of nursing activity in the original colonies and worker tasks, we also examined the effect on the task choice of nurses in the nurse-biased colonies (Fig. 1b). In nurse-biased colonies, we also found that nurses in the original colony shifted to foragers depending on the nursing activity in their original colony (GLMM: *χ*^2^ = 25.84, *P* < 0.001, *R*^2^ = 0.924; Fig. 2b, Additional file 1: Table S1). We also found the conversion rate of nurses (37/490 nurses) was lower than those of foragers (105/391 foragers) (*χ*^2^ test with Yates correction: *χ*^2^ = 195.32, *P* < 0.001). Thus, our results suggest that under disturbed conditions, workers switched from their original task to the other task. While the effect of the recent behavioural pattern was clearly evident in the case of foragers, it should be noted that a small number of nurses switched to the forager task in the nurse-biased colonies.

### Effect of task experience on task choice

**Fig. 3:**
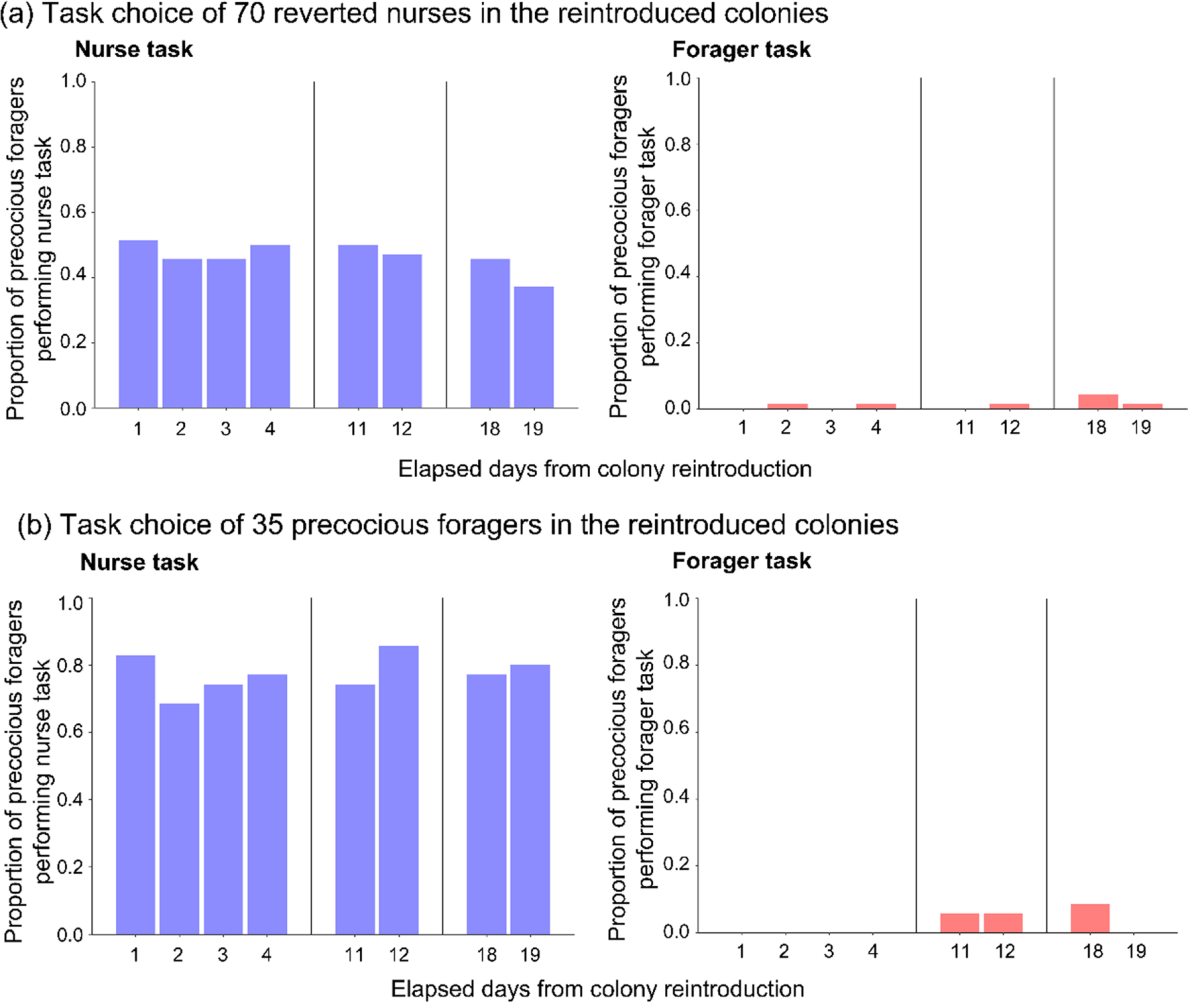
Proportions of workers that performed forager-task or nurse-task in the reintroduced colonies. Most **a** reverted nurses and **b** precocious foragers did not perform the forager-task but the nurse-task instead. Blue and red bars indicate the proportion of workers that performed the forager- and nurse-task, respectively

To evaluate the effect of experience on task choice, we examined the task choice of reverted nurses (original foragers that switched to nurse-task) in the reintroduced colonies. We found that the interaction between the treatment and the number of elapsed days was statistically insignificant in either tasks of reverted nurse and precocious forager (GLMM, reverted nurse: nurse task: *χ*^2^ = 0.198, *P* = 0.657, *R*^2^ = 0.047; forager task: *χ*^2^ = 0.400, *P* = 0.527, *R*^2^ = 0.134; precocious forager: nurse task: *χ*^2^ = 1.732, *P* = 0.188, *R*^2^ = 0.280; forager task: *χ*^2^ = 0.284, *P* = 0.594, *R*^2^ = 0.831; Fig. 3, Additional file 1: Table S2). Therefore, we combined data from both treatments. In the reintroduced colonies, about half of reverted nurses continued nursing (mean proportion ± SD: 0.47 ± 0.04), whereas almost none returned to foraging (0.01 ± 0.01). Additionally, our analysis indicated that reverted nurses were significantly more likely to choose the nurse task regardless of time elapsed after reintroduction (GLMM: task: *χ*^2^ = 104.818, *P* < 0.001, *R*^2^ = 0.591; days: *χ*^2^ = 0.753, *P* = 0.386, *R*^2^ = 0.021; interaction: *χ*^2^ = 3.268, *P* = 0.071, *R*^2^ = 0.741; Fig. 3a, Additional file 1: Table S3). Next, we conducted the same analysis for precocious foragers (original nurses that switched to forager-task); we expected that task experience would decrease their response thresholds to the foraging task. In stark contrast to reverted nurses, the task choice (nurse task: 0.78 ± 0.05; forager task: 0.03 ± 0.04) was biased towards nurse task (GLMM: task: *χ*^2^ = 121.481, *P* < 0.001, *R*^2^ = 0.686; days: *χ*^2^ = 1.502, *P* = 0.220, *R*^2^ = 0.015; interaction: *χ*^2^ = 2.868, *P* = 0.090, *R*^2^ = 0.676; Fig. 3b, Additional file 1: Table S3). Note that all data are summarized in Additional file 1: Table S1–S3.

**Fig. 4:**
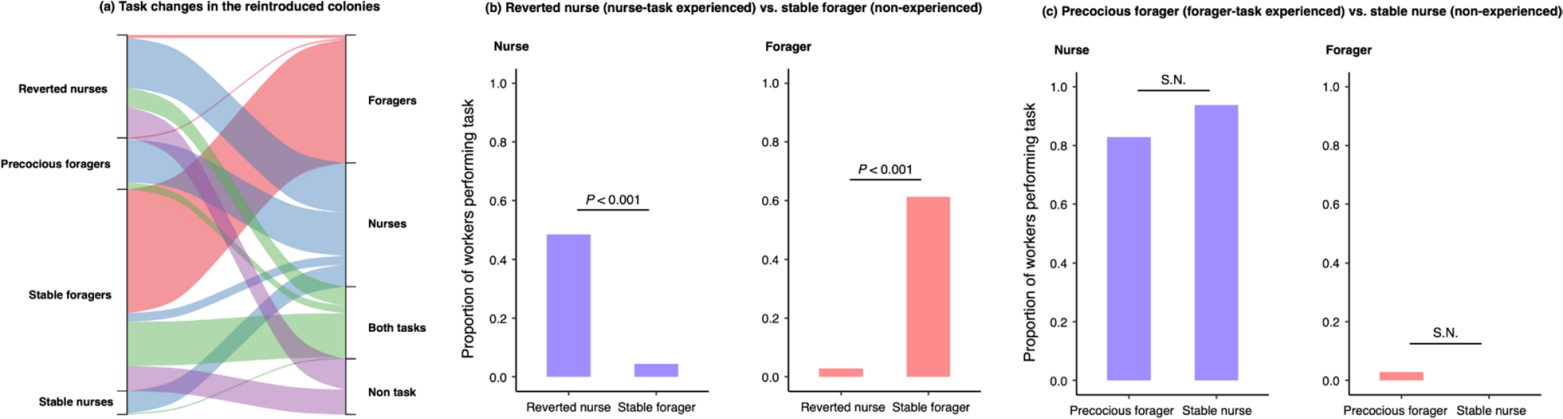
Proportion of workers categorised into forager or nurse in the reintroduced colony. **a** Task changes of all workers in the reintroduced colonies **b** Comparison of the proportion of forager and nurse between reverted nurses (nurse task experienced foragers) and stable foragers (non-experienced nurses). **c** Comparison of the proportion of forager and nurse between precocious foragers (forager task experienced nurses) and stable nurses (non-experienced nurses). Blue and red bars indicate the proportion of workers that performed the forager and nurse task, respectively

Next, we strictly validate the effect of task experience on threshold change. Before testing, we categorized workers into three types using scan observations during 8 days after the colony fusion (Fig. 4a; Table 1). If workers shifted to other tasks from their original ones in the reintroduced colonies, it can be considered that their threshold against the tasks changed through task experience (Fig. 1c). We found that the proportion of reverted nurses performing nurse task was higher than those of the stable foragers (Fisher exact test, *P* < 0.001; Fig. 4b), while the opposite was true for the forager task (Fisher exact test, *P* < 0.001; Fig. 4b). However, in case of precocious foragers (forager-task experienced nurses) and stable nurses (non-experienced nurses), there was no significant statistical difference in the proportion of workers performing forager task or nurse task (Fisher exact test, nurse task: *P* = 0.413; forager task: *P* = 1; Fig. 4c). These results strongly suggest that the effect of task-experience decreased the threshold of foragers against the nurse task. Note that there was a possibility that the low proportion of broods to workers, prevents workers from enhancing the nurse task experience, leading to low efficiency of reinforcement. Therefore, we considered that the reverted nurse in the low-proportion colonies tends to stop performing the nurse task in the reintroduced colonies. However, our results suggest that the proportion had no effect on the ratio of reverted nurses that stopped performing the nurse task (*χ*^2^ test after GLM with Poisson error distribution: larvae: *χ*^2^ = 0.427, *P* = 0.513, *R*^2^ = 0.084; eggs: *χ*^2^ = 0.152, *P* = 0.696, *R*^2^ = 0.021; larvae and eggs: *χ*^2^ = 0.251, *P* = 0.616, *R*^2^ = 0.038). Therefore, it has been considered in this experimental design that, the ratio of broods to individuals had no contribution to individual experience of the worker.

**Table 1:** Number of workers categorised into each type in the reintroduced colonies

Original task	Task in the biased colony	Categorised tasks in the reintroduced colony			
		Forager	Nurse	Both tasks^a^	Non-task^a^
Forager	Reverted nurse	2	34	13	21
	Stable forager	84	6	30	17
Nurse	Precocious forager	1	29	5	0
	Stable nurse	0	15	1	0

^a^Both categories are defiend as "undefined" (see Fig. 1a)

## Discussion

We obtained three main results in this study on the behavioural flexibility of *D.* cf. *indicum* workers under artificially disturbed conditions. First, we confirmed the behavioural flexibility of workers. Some foragers became reverted nurses, shifting to the nurse-task when nurses were removed from the colony, while nurses also shifted to forager-task as precocious foragers when foragers were removed (Fig. 2). Second, we found that reverted nurses did not resume foraging, but continued nursing even after they were merged with nurses in the original colonies. Third, in contrast to the reverted nurses, about half the precocious foragers resumed the nurse-task and only a few precocious foragers continued to the forager-task in reintroduced colonies (Fig. 3). It was observed that the nurse task experience strongly contributed to the task change of the original foragers, while the forager task experience had no effect on the original nurses.

Worker behavioural flexibility enables a colony to allocate limited workforce to a focal task depending on demand [43]. Combined with results from previous studies [15, 19], our findings suggest that *D*. cf. *indicum* workers generally exhibit flexibility in task shifting. In the field, forces such as nest relocation frequently cause their colonies to face biased age structure [39, 40, 42], and divided colonies are observed to fuse in *D.* cf. *indicum* (H. Shimoji, personal observation) and in the related ant species *D. indicum* [44]. In these fluctuating environmental conditions, the bidirectional task shifting that we observed is expected to cause a prompt DOL reconstruction.

A previous study showed that when *D*. cf. *indicum* nurses were removed from a colony, partial foragers shifted to the nurse as a specialist role [15]. Because a fixed response threshold could explain these task reversions, with the foragers that become reverted nurses possessing a lower initial threshold than other foragers, it is difficult to determine whether task reversion is actually the result of a change in forager threshold. In this study, some reverted nurses maintained their nursing task and rarely foraged once the original nurses (precocious foragers and stable nurses) were merged into the colony (Fig. 3). If the response thresholds of reverted nurses were fixed, we would expect all reverted nurses to stop nursing and forage instead, because the threshold of original nurses may be lower than reverted nurses (see “Method” section). In contrast to the fixed threshold hypothesis, our results suggest that the threshold of foragers changed in response to their individual experience with the nurse-task, leading to task specialization in the disturbed colony. While previous studies have suggested that past experience affects worker task choice [11, 32], here we performed a strict test on the effect of individual experience [33] and provided a strong evidence of task specialization caused by a change in response threshold, at least among reverted nurses.

Tripet and Nonacs [11] also tested the effect of task experience on the task choice using the carpenter ant, *Camponotus floridanus*. They combined categories consisting of minor workers, i.e., the precocious foragers and the stable nurse, or the reverted nurse and the stable nurse. After a short observation period (3 days after manipulation), they found that the task choices were ruled to their recent experience. Although the experimental design was different from our study in that they used sub-colonies consisting of only workers and almost all workers were infertile under their experimental design (see also [45]), they also revealed the effect of task experience on task choice (see also later part for the different results of the reverted nurses are described towards the end of this section). Therefore, the effect of task experience on the task-choice of workers might be conserved across ant taxa.

Precocious foragers returned to the nurse-task in the reintroduced colonies (Fig. 4a), suggesting that social interaction facilitated the forager task in nurse-biased colonies. In some species including *D*. cf. *indicum*, a dominance hierarchy formed through repeated interactions promotes reproductive DOL [46]. Generally, dominance interactions occur frequently in queen-absent colonies, with dominant workers able to reproduce directly [47–49]. Given the known presence of dominance hierarchies in *D*. cf. *indicum*, we propose the following behavioural mechanisms for facilitating the forager task in a nurse-biased colony. First, the dominance hierarchy among the nurses was established in the nurse-biased colony. In *D.* cf. *indicum*, dominant workers are not involved in the tasks outside the nest [49], resulting in subordinate nurses becoming precocious foragers in a queen-absent colony. In contrast, because dominant workers were absent in the forager-biased colony, precocious nurses returned to nursing in the reintroduced colony. Interestingly, the proportion of reverted nurses who quit performing the nurse task in the reintroduced colonies did not depend on the time spent on the nurse task in the forager-biased colonies (see text in Additional file 1: Fig. S4). This result may imply that dominance interaction also affects the task choice of reverted nurse in the reintroduced colonies. Our data thus suggest that dominance hierarchies may play a role in DOL reconstruction of this ant species.

Many precocious foragers shifted back to the nurse-task after reintroduction, regardless of the last task experienced, implying that nurses persist in their original task (Fig. 3). This result seems to be inconsistent with the case of reverted nurses that returned to the forager task. Our findings may indicate that task experience has a differential effect on the response threshold of original foragers versus original nurses. In general, the physiological states of workers are modulated to the specific tasks [18–22, 50–55]. Additionally, some studies have evaluated physiological changes of workers that have been “re-specialized” to a specific task. In the honeybee, for example, the brains of task-shifted workers have similar physiological states as those of workers originally performing the tasks [20]. In the ant *Temnothorax longispinosus*, worker gene-expression patterns are modulated to their task rather than to age or fertility [56]. Similarly, brain *Vg* and *Vg-like A* expression plays a role in regulating the tasks of several insect species (e.g., [18, 57]). In *D.* cf. *indicum*, after reverted foragers have nursed for at least 7 days, they exhibit upregulation of brain *Vg* and *Vg-like A* to the same level as original nurses [19]. Therefore, we suggest that original nurses require more time for physiological modulation than original foragers, resulting in the differential impact of task experience. Further study focusing on temporal shifts in the physiological states of precocious foragers will improve our understanding of the relationship between response threshold and individual experience.

Differences between foragers and nurses in their response to task experience also have ultimate mechanisms. In this species, nurses (generally young workers) have active ovaries and worker reproduction occurs in the colony, whereas foragers (generally old workers) have lost most of their reproductive capacity [49, 58, 59]. Moreover, a previous study showed that abdominal *Vg* and *VgR* expression levels in reverted nurses remained as low as in the stable foragers, suggesting that their reproductive capacity has not been restored [19]. Therefore, if nurses maintained active ovaries even while working as precocious foragers, they could improve their direct fitness through returning to the nurse-task within the nest. In contrast, because reverted nurses have no active ovaries, they should improve indirect fitness through helping relatives, performing tasks depending on colony demands. Interestingly, in *C*. *floridanus*, the effect of task-experience affected both young and old minor workers that did not possess developing ovaries [11]. Therefore, these asymmetric fitness gains (varying reproductive ability) could modulate intrinsic parameters such as degree of reinforcement, leading to task experience exerting different effects on task choice among workers.

## Conclusions

Flexibility in shifting worker tasks is crucial to maintaining DOL under fluctuating environmental conditions [8]. In this study, we followed the methods of a previous study to perform manipulation experiments [33] that tested whether task experience shifted a worker’s response threshold, resulting in task flexibility. We clearly showed that task experience would alter forager response thresholds, but our data did not support this effect on nurses. Future research should aim to clarify the physiological basis of variation in responses to individual experience. Based on our findings, we speculate that differences in the experience effect between foragers and nurses may reflect distinct strategies of increasing fitness. Although some studies have attempted to investigate evolution of DOL in terms of response threshold as a target of natural selection [60–62], we believe that further theoretical advances along these lines will improve our understanding of DOL.

## Methods

### Collection and rearing of *Diacamma* cf. *indicum*

We collected *D*. cf. *indicum* colonies from the main island of Okinawa during July and September 2020. A colony consists of the gamergate and 50–300 workers [39, 40]. In this species, all emerged workers possess a pair of tiny appendages, called gemmae, on the thorax [38]. The gamergate bites off the gemmae of newly emerged individuals, causing them to irreversibly differentiate into the worker caste.

We subjected eight colonies with gamergates to behavioural experiments (colony A: 76 workers, colony B: 195 workers, colony C: 175 workers, colony D: 134 workers, colony E: 199 workers, colony F: 181 workers, colony G: 129 workers, colony H: 130 workers). Each worker was marked with a unique colour pattern using enamel pens. Colonies were then maintained in plastic containers (240 mm × 180 mm × 85 mm) including an artificial nest (148 mm × 84 mm × 32 mm) until experiments began. Throughout the experiments, ants were fed standard artificial diets (1:1 ratio of protein to carbohydrate) [63] and fresh mealworms three times per week, given water daily, and kept in the laboratory at 25 ± 1 °C with a 14:10 light:dark photoperiod.

### Behavioural observations

Colonies were transferred into experimental nests (148 mm × 84 mm × 32 mm or 221 mm × 141 mm × 37 mm) connected to a foraging area (240 mm × 180 mm × 85 mm) at least 1 day before beginning behavioural observations. Scan sampling was employed to categorize worker tasks, with digital cameras (DSC-WX350, SONY, Tokyo, Japan; iPhone XR, Apple, Cupertino, CA, USA) taking photographs of the artificial nest and foraging area 10 times per day, with intervals of at least 30 min between observations. Worker location was recorded based on these photographs. Workers were categorized into three types: physical contact with brood items (nursing), outside the nests (foraging), or neither. After recording states for all workers in each observation period, we classified individuals as nurse or forager according to our previous study [15]: We defined workers performing only nursing at least once during the observation period as nurses (Fig. 1a), and those that performed only foraging at least once during the observation period as foragers (Fig. 1a). Workers that engaged in neither or both tasks were classified as an undefined worker and excluded from further analyses (Fig. 1a).

### Reintroducing experiments

A previous model [33] suggests a series of manipulation experiments to test the change of threshold by task experience as follows: Nurses are removed from a colony, and then the reverted nurses are induced in the colony; the removed nurses prevented from performing the nurse task are re-introduced to the original colony; the researchers evaluate whether the reverted nurses continue the nurse task or not. We summarized the interpretations in results according to the prediction of the model (Fig. 1c). In addition to the above observation, we examined the task choice of all workers in the reintroduced colonies.

First, to define worker tasks in an unmanipulated (original) colony, we conducted 40 behavioural observations over four consecutive days (10 times per day). Based on the 40 observations, we categorised the workers into three types (Fig. 1a). Within 1 day after observations ended, the original colony was split into a nurse-biased (only nurses) and a forager-biased (only foragers) colony (Fig. 1b). Both sub-colonies contained the same number of eggs and larvae. Gamergates were added to the forager-biased colonies only. After the manipulation, we performed 4 days of scan observation daily (40 behavioural observations over four consecutive days) to determine worker tasks during DOL reconstruction (Fig. 1b). Based on the above observations, we categorised workers into three types using the 40-observation period, and finally obtained 391 foragers, 490 nurses, and 338 undefined workers (both tasks: 124 workers; non task: 214 workers). Workers that engaged in neither or both tasks were classified as an undefined worker and excluded from further analyses (Fig. 1a). Note that in this species, long-time isolation from the gamergate causes several physiological changes in workers, leading to behavioural changes (e.g., [49]). Thus, we added gamergates to minimize the impact on the physiological changes of workers.

Next, to test the effect of experience on task choice, we moved some workers from the nurse-biased colony to the forager-biased colony (Fig. 1b). Workers selected for the move were those that remained nurses (hereafter, stable nurse) and those that became foragers (hereafter, precocious forager) or only the precocious foragers. From the forager-biased colony, we also removed the same number of nurses (hereafter, reverted nurse) and foragers (hereafter, stable forager) as the number of collected workers in the nurse-biased colony. This change allows all workers in the reintroduced colony to select any task. To set up this experiment, we transferred eggs (mean ± SD: 21.1 ± 4.1), larvae (mean ± SD: 4.8 ± 2.5), and the gamergate from the forager-biased colony to a new artificial nest. All selected workers from both sub-colonies were then released into the foraging area connected with this new nest. In this experiment, we prepared two types of treatments. First, using colonies A, B, C, and D, precocious foragers (mean ± SD: 5.3 ± 3.0) were reintroduced into the forager-biased colony (treatment1; Fig. 1b). In this treatment, the task-choices of the stable nurses in the reintroduced colonies are interpreted as the control, because the threshold of the stable nurse would be expected to stabilise. Next, to confirm the task choice of stable nurses, we reintroduced precocious foragers (mean ± SD: 4 ± 1.4) and stable nurses (mean ± SD: 4 ± 1.4) into the forager-biased colony using colonies E, F, G, and H, (treatment2; Fig. 1b). In both treatments, the task choice of stable foragers can also be regarded as control. We observed the reintroduced colonies on days 1–4, 11–12, and 18–19 (10 times per day). In these experiments, we categorised worker types daily based on 10 observations. In addition, to validate the effect of task experience on threshold change, we categorised all workers in the reintroduced colonies based on 80 observation periods. If task experience has no effect on the response threshold of workers, we predicted that task-shifted workers in the sub-colonies would return to the tasks they had in their original colony (Fig. 1c). If individual experience sufficiently reduces response thresholds, we predicted that task-shifted workers would continue their current tasks (Fig. 1c). Note that for one colony (colony G), no workers were removed because the number of workers in the forager-biased colony was too low, and another colony (colony A) was excluded from the reintroducing experiment because all reverted nurses died within 4 days in the reintroduced colony.

### Statistical analysis

#### Effect of behavioural propensity on task choice

Our previous study showed that forager frequency in the original colony was correlated with the tendency to task shift [15]. To confirm this result, we examined how proportion of time (to total observation time) spent in contact with the brood (nurse-task) or outside the nest (forager-task) in the original colony was related to worker tasks in forager-biased and nurse-biased colonies. We used the R package glmmTMB [64] to construct generalized linear mixed models (GLMMs) with a binomial error distribution. As the response variable (task), nurses and foragers in the sub-colonies were binarised to 0 and 1, respectively. The proportion of foragers or nurses across the total observation period (40 observations) in the original colony was the fixed effect, and colony ID was the random effect.

#### Effect of task experience on task choice

To further understand the effect of individual experience on task choice, we focused on reverted nurses after reintroducing workers from the nurse-biased colony to the forager-biased colony. We first used a GLMM with a binomial distribution to compare the proportion of reverted nurses that performed the nurse or forager task in treatment 1 with those in treatment 2. The number of elapsed days, treatment, and their interaction were set as fixed effects. We set the proportion of individuals performing focal tasks (forager or nurse) in each sub colony as a response variable. Colony ID was set as a random effect. Because none of the fixed effects significantly influenced either task in reverted nurses (see “Results” section), we included all days and treatments in our GLMM to compare task selection (forager or nurse) among reverted nurses. We set task as a fixed effect, performing/not performing the focal task as the response variable, and colony ID as a random effect. This analysis included only reverted nurses that survived until day 19 after reintroduction (treatment 1, *n* = 35; treatment 2, *n* = 35).

In this study, *P* values of fixed effects were evaluated with the *Anova* function (Type II ANOVA) of the car package. Effect sizes of the models were determined with conditional *R*^2^ values [65] using the *r.squaredGLMM* function of the MuMIn package [66]. All constructed models were checked for overdispersion using the *testDispersion* function of the DHARMa package [67]. Statistical analyses were performed in R version 4.0.2 [68].

## Supplementary Information

**Additional file 1**. Supplementary Information.

## Data Availability

All data using this study are deposited in the Additional file 1.
