# Supplementary material for: Individual experience influences reconstruction of division of labour under colony disturbance in a queenless ant species
Source: Front Zool. 2022 Jun 15;19:20. doi: 10.1186/s12983-022-00466-9 (PMC9202139; doi:10.1186/s12983-022-00466-9)
Supplement: Supplementary file 1 — Additional file 1. Supplementary Information. [file 12983_2022_466_MOESM1_ESM.docx]

Supplementary Information of

Individual experience contributes to reconstruction of the division of labour in an ant

Yasunari Tanaka, Masaru K Hojo, Hiroyuki Shimoji^†^

School of Biological and Environmental Sciences, Kwansei Gakuin University, Sanda, Hyogo 669-1337, Japan

^†^Corresponding author: shimojih@kwansei.ac.jp

**Results**

**Task transition of workers in the disturbed condition**

We also examined the relationship between the behavioural propensity in the original and sub-colonies. In the forager-biased colony, the foraging activity was positively correlated with foraging activity in the original colony (GLMM: *χ*² = 143.93, *P* < 0.001, *R*² = 0.087; Fig. S1, Table S4), whereas the nursing activity was negatively correlated (GLMM: *χ*² = 627.11, *P* < 0.001, *R*² = 0.121). In addition, the nursing activity in the nurse-biased colony was positively correlated with that of the original colony (GLMM: *χ*² = 75.825, *P* < 0.001, *R*² = 0.022; Fig. S1, Table S4) and negatively correlated with the foraging activity (GLMM; *χ*² = 38.615, *P* < 0.001, *R*² = 0.053).

**Change in frequency of nurse task in reverted nurses in the reintroduced colonies.**

To examine whether the nurse task frequency changes in the reintroduced colonies, we compared the proportion of time spent on performing nurse task in the forager-biased and the reintroduced colonies. 46 reverted nurses performed the nurse task after reintroducing treatment, while 24 reverted nurses never performed the nurse task. We found that there was no effect of the recent task frequency on nurse task in the reintroduced colonies (Wilcoxon signed rank test, *W* = 587, *P* = 0.171; Fig. S4a). Moreover, the reverted nurses stopped performing the nurse task independent of the recent nurse-task frequency (Fig. S4b). These results suggest that other factors can also affect the task choice in reintroduced colonies (see Discussion).


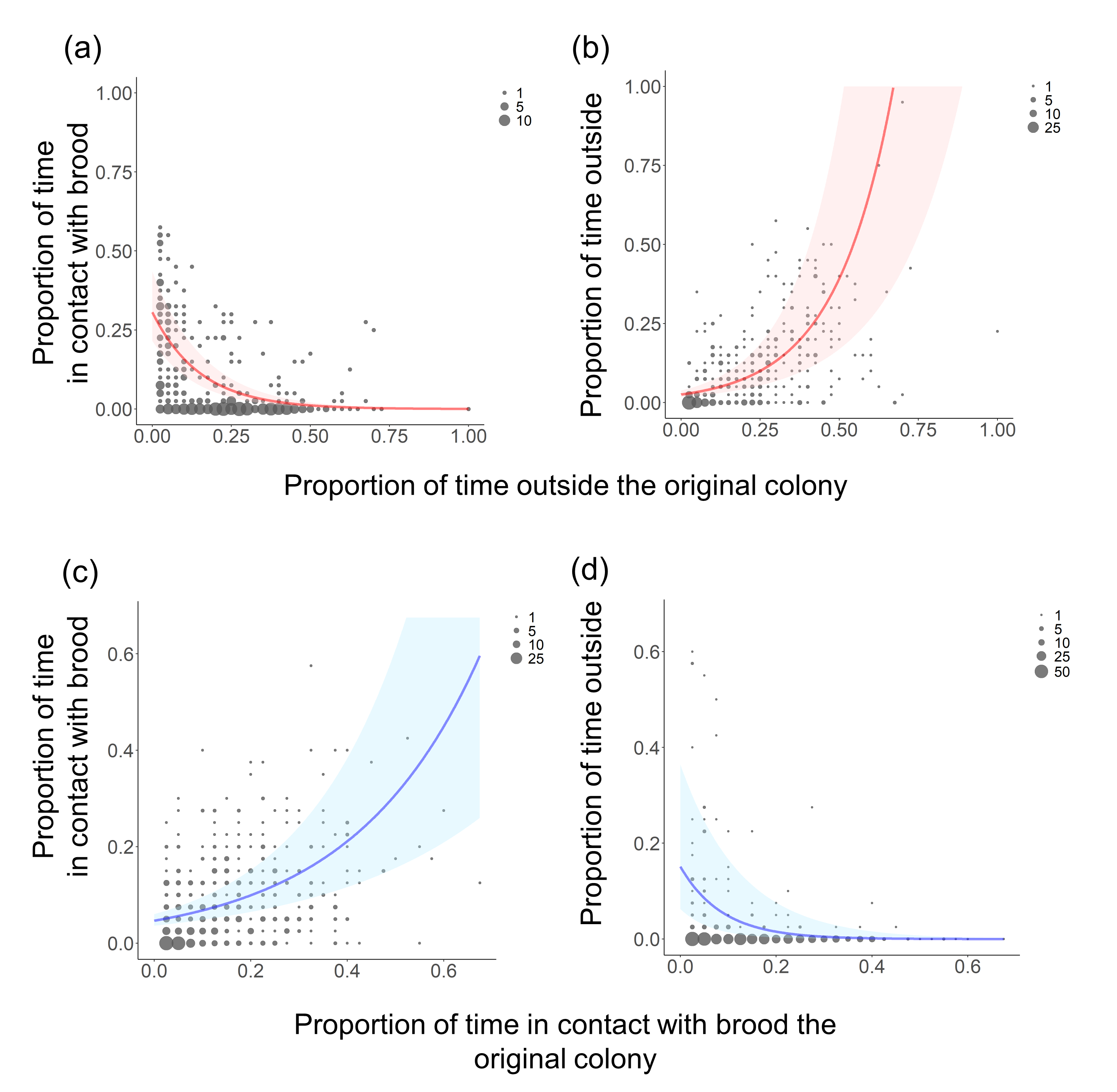


Figure S1. The relationships between behavioural propensity in the original colony and sub-colonies: (a, b) forager-biased colony and (c, d) nurse-biased colony. Circle sizes represent the number of overlapping data points. Lines and shading indicate estimated prediction and 95% confidence intervals, respectively. GLMM: (a) *χ*² = 627.11, *P* < 0.001, *R*² = 0.121; (b) *χ*² = 143.93, *P* < 0.001, *R*² = 0.087; (c) *χ*² = 75.825, *P* < 0.001, *R*² = 0.022; (d) *χ*² = 38.615, *P* < 0.001, *R*² = 0.053.





Figure S2. The proportions of each caste categorized to each task in the reintroduced colony (treatment 1).


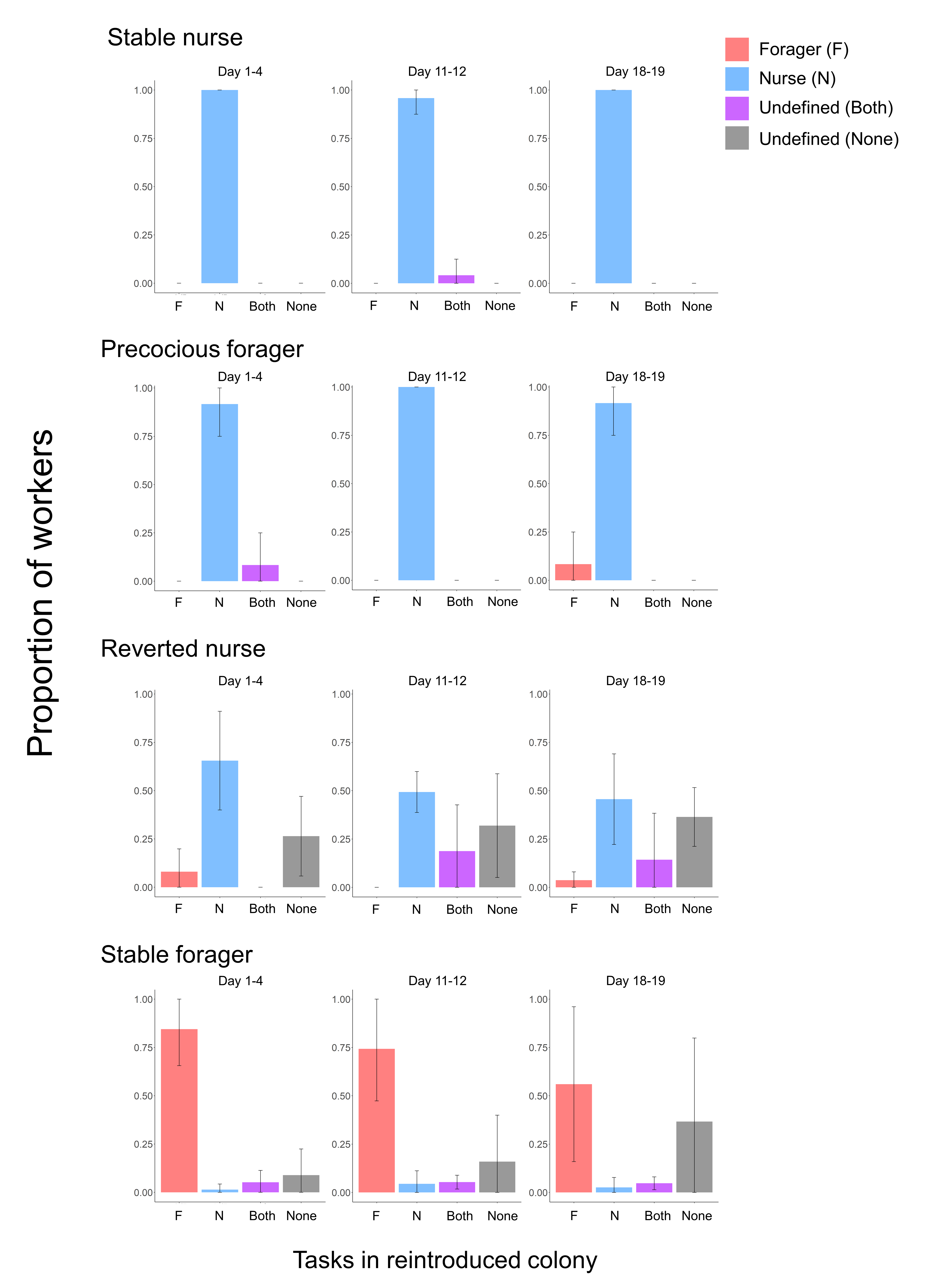


Figure S3. The proportions of each caste categorized to each task in the reintroduced colony (treatment 2).


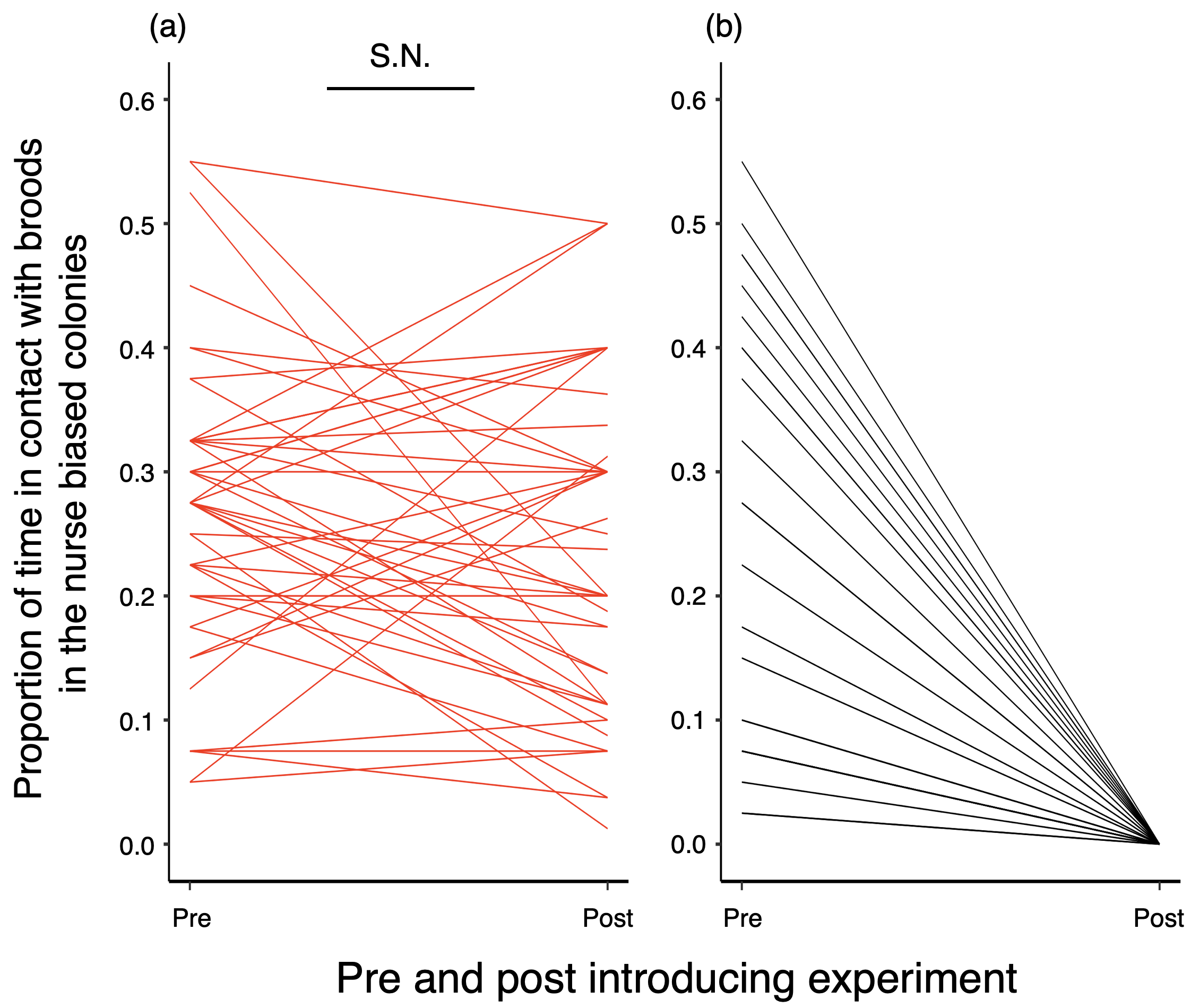


Figure S4. Change of proportion of time spent on performing nurse-task before and after introducing experiment. (a) Comparison of the proportion of time spent on performing the nurse task in reverted nurses who continued nurse task in the reintroduced colony. (b) Some reverted nurses stopped performing nurse task in the reintroduced colonies.
